# Toxic effects of *Lepidium sativum* seed fixed oil on Wistar albino rats in acute and subacute toxicity models

**DOI:** 10.3389/ftox.2025.1535597

**Published:** 2025-03-21

**Authors:** Teshome Gebremeskel Aragie, Kaleab Asres, Wondwossen Ergete, Samual Woldekidan, Sileshi Degu, Abiy Abebe, Worku Gemechu, Derso Furgasa, Girma Seyoum

**Affiliations:** ^1^ Department of Anatomy, College of Health Sciences, Woldia University, Woldia, Ethiopia; ^2^ Department of Anatomy, College of Health Sciences, Addis Ababa University, Addis Ababa, Ethiopia; ^3^ Department of Pharmaceutical Chemistry and Pharmacognosy, College of Health Sciences, Addis Ababa University, Addis Ababa, Ethiopia; ^4^ Department of Pathology, School of Medicine, College of Health Sciences, Addis Ababa University, Addis Ababa, Ethiopia; ^5^ Biomedical and Clinical Research Team, Traditional and Modern Medicine Research Directorate, Armauer Hansen Research Institute, Addis Ababa, Ethiopia; ^6^ Clinical Chemistry Department, Clinical Trial Research Directorate, Armauer Hansen Research Institute, Addis Ababa, Ethiopia

**Keywords:** *Lepidium sativum*, seed fixed oil, Wistar rats, acute toxicity, subacute toxicity

## Abstract

**Introduction:**

*L. sativum* L. (family Brassicaceae) is a versatile herbal medicine in Ethiopia. The seed extract is widely employed in traditional medicine, whilst the seed oil is used as edible oil. However, there are no available studies conducted on the safety of the fixed oil of *L. sativum* seed in Ethiopia. Therefore, this study aimed to evaluate the acute and subacute toxicity of the oil in Wistar albino rats.

**Methods:**

Acute and subacute toxicity studies were conducted in Wistar albino rats. A single oral dose of *L*. *sativum* seed oil was administered, and the animals were followed for 14 days. The subacute oral dose toxicity study was conducted in rats of both sexes by repeated 28-day toxicity test as per OECD guidelines. Body weight was measured weekly, and observations of the animals were made regularly throughout the study period. Organ weight, histopathology, hematology, and clinical chemistry data were collected on the 29^th^ day. One-way analysis of variance (ANOVA) was used to compare the means of the comparison groups and the results were presented as mean ± standard deviation, and significance was determined at the P-value of <0.05.

**Results:**

In this study, the LD_50_ of the fixed oil of *L. sativum* was found to be 2818.32 mg/kg. According to the World Health Organization, the oil is classified as slightly hazardous at a single oral dose administration. In the subacute toxicity study, rats treated with the oil showed significant changes behavioral indices such as piloerection, lethargy, and tremor. In addition, gross pathology of organs, body weight, biochemical, and hematological parameters were deranged.

**Conclusion:**

The results of the present study demonstrated that the fixed oil of *L. sativum* has toxic effects. Therefore, it is highly essential to create awareness among the Ethiopian public who use the seeds for medicinal purposes and/or consume the oil as edible oil about the possible health hazards that they may pose.

## Introduction

Natural products were practically the only treatment option for diseases that afflicted humanity before the advancement of pharmacological treatments (Costa et al., 1998; Yunes et al., 2001). Interest in natural products i.e., herbal medicines has exponentially increased in recent decades (Akbar, 2020). Especially an overwhelming majority of the rural population in the world still relied on plant-based drugs for their healthcare need (Smith-Hall et al., 2012). Although it is generally believed that most herbal preparations are safe for consumption, it should be noted that they contain xenobiotic agents, where their biotransformation products can be potentially toxic (Lapa et al., 1999).

Globally, among thousands of medicinal plants, *Lepidium sativum* (Brassicaceae) is a well-known medicinal plant widely consumed across different continents. In Ethiopia, it is called *“Feto”* in Amharic, cultivated for its medicinal value, and edible oil is produced from its seed. Some researchers claim that it originated in Ethiopia and then distributed to various parts of the world, while others say that it started from southwest Asia and then spread to Western Europe (Falana et al., 2014; Sharma et al., 2011).

The seed is brown to brownish red 2–3 mm in size, has an oval shape with a smooth surface, and a bitter taste (Ahmad et al., 2015). It possesses varied medicinal values, known as a “versatile medicine” in Ethiopia (Teklehaymanot et al., 2007). Ethnomedicinally *L. sativum* seeds are consumed in the form of a special plate known as “*Feto Fitfit”* which is a mixture of ground seed powder, water, salt, lemon, and pieces of injera (Ethiopian bread similar to traditional pancake) to relieve backache and to cure diarrhea (Gilani et al., 2013), abdominal pain, dysentery, and parasitic worm infestation (Hussain and Ghani, 2008; Gupta et al., 2010). It is also used as a food supplement in the human diet as it contains a considerable number of vitamins and minerals such as iron and calcium. High carbohydrates, macro and microelements, and antioxidant properties would also increase its recognition as a functional food (Sheel Sharma et al., 2011; Kasabe et al., 2012).

*L. sativum* seed contains 24% oil which is composed mainly of α-linolenic acid (ALA) (32%) and linolenic acid (LA) (12%). This oil is reactively stable owing to its high content of antioxidants and phytosterols (Diwakar et al., 2010). The seed oil of *L. sativum* has coagulating property (Maheswaraiah et al., 2018), antioxidant, anti-microbial, and anti-inflammatory effects (Alqahtani et al., 2019; Abo El-Maati et al., 2016).

The safety of *L. sativum* was studied using its extracts, however, evidence on the safety of the seed’s fixed oil is still limited. Therefore, this study aimed to evaluate the acute and sub-acute toxicity of *L. sativum* seed oil in Wistar Albino rats.

## Materials and methods

### Study setting and experiment

The study was conducted in the Ethiopian Public Health Institute (EPHI), Modern and Traditional Medicine Department laboratory, Addis Ababa, Ethiopia, from January 2024 - March 2024.

### Collection of plant material and preparation of extract

Seeds of *L. sativum* were collected from in and around Woldia town, 521 km Northeast of Addis Ababa, Ethiopia. Authentication of the plant was carried out by the National Herbarium, Department of Biology, College of Natural and Computational Sciences, Addis Ababa University where a voucher specimen (collection number TG 001) was deposited for future reference. The seeds were cleaned, washed, dried, and ground to powder using an electric mill and stored at room temperature. The powder was mixed with *n*-hexane in a 1:10 ratio of powder to solvent in Erlenmeyer flasks wrapped in aluminum foil and subjected to extraction in an orbital shaker for 24 h. It was then filtered using Whatman No 1 filter paper (Merck, Darmstadt, Germany). The organic solvent was removed using a rotary evaporator (Büchi R-205, Switzerland) at 40 °C and 175 millibar pressure. The oil obtained was kept in a wrapped glass bottle and stored in a refrigerator at −20°C until used (Zhang et al., 2018).

### GC-MS analysis of *Lepidium sativum* fixed oil

Before conducting the GC-MS analysis, the esterification of the fixed oil was performed as follows: A sample of 0.2 g of *L. sativum* oil was added with 3 mL of 2N KOH in methanol and refluxed for 1 hour. After refluxing, the mixture was allowed to cool to room temperature, and then 5 mL of 5% HCl in methanol was added. The mixture was refluxed again for an additional hour. Once the second reflux period was complete, the mixture cooled to room temperature. It was then transferred to a separatory funnel, where an equal volume of N-hexane and 3 mL of water were added. The aqueous phase was discarded, and the organic phase was carefully collected into GC-MS vials. Finally, the samples were injected into the GC-MS for analysis, ensuring a precise and thorough evaluation of the results.

GC-MS analysis was performed using a GC (Agilent Technologies 7890B, United States) coupled with a mass spectrometer (Agilent Technologies 5977A Network). The GC was equipped with an HP-5MS non-polar column (Agilent Technologies), measuring 30 m in length with a 250 μm internal diameter and a film thickness of 0.25 μm. Helium was used as the carrier gas, flowing at a 1 mL/min rate. The injector temperature was set to 250°C, and the injection mode was configured to split mode with a split ratio of 30:1. The initial oven temperature was programmed to start at 80°C, held for 2 min, and then increased to 200°C at a rate of 10°C/min. Following that, the temperature was ramped up at 5°C/min until it reached 250°C, before increasing to 280°C at a rate of 15°C/min, where it was maintained for 9 min. Mass spectra were recorded in electron impact (EI) mode at an energy level of 70 eV, scanning within the 50–550 m/z range.

### Experimental animals

Healthy, nulliparous Wistar albino rats, weighing 180–220 g, and ages 10–12 weeks were used for all the experiments. The animals were obtained from the EPHI animal breeding unit. They were kept in the animal house of the Traditional and Modern Medicine Research Directorate of the EPHI and acclimatized to the environment for 1 week before the commencement of the actual experiment. The animals were placed in stainless steel cages in an environmentally controlled room with temperature (23°C ± 3°C), relative humidity (50% ± 10%), and 12 h light and dark cycles. During the adaptation period, all animals were fed a standard pellet (composed of carbohydrate (75%), protein (16%), fat (5.5%), calcium (3.6%), and phosphorus (0.4%)) with free access to tap water (Suzuki, 2021).

### Experimental model

#### Acute toxicity and medial lethal dose (LD_50_) of fixed oil of *L. sativum*

Acute toxicity study was performed in female rats in a stepwise procedure using 5 animals per step as recommended by OECD 423 guidelines (No, 2002). A total of 30 nulliparous non-pregnant female albino Wistar rats, age range between 8–12 weeks were divided into six groups (GI, GII, GIII, GIV, GV, and GVI), each group having 5 animals. All the animals, test groups (GI, GII, GIII, GIV, and GV), and control group (GVI) were restricted from food and water for 12 h (overnight) before administration and their body weight was recorded (OECD, 2016). The test preparation for the experimental group and distilled water for the control group were calculated. Then, oil was administered as a single oral dose using a suitable intubation cannula starting with 300 mg/kg, and the next animal group was dosed based on the response. Animals were restricted from food for 3–4 h and observed for any behavioral change for the first 4 h following the administration for any toxicity manifestation like changes in skin and fur, eyes and mucous membranes, respiratory, circulatory, autonomic central nervous systems, and somatomotor activity behaviors were observed. More attention was given to severe signs of toxicity like increased motor activity, piloerection, salivation, convulsion, coma, and death. Subsequent observations were made at regular intervals for 24 h (OECD, 2008). The animals were kept under further follow-up for 14 days at least for 2 h per day. The times at which signs of toxicity appear and disappear were noted, especially if there was a tendency for toxic signs to be delayed (OECD, 2008) and the number of rats that died within the study period was recorded.

The body weight of the rats was recorded on the 1^st^ day (before administration), 7^th^, and 14^th^ days of the experiment. Any difference in body weight of each rat was recorded by taking the difference from the initial (weight before administration). On the 14^th^ day of the study, the final weight of each rat was recorded after about 12 h of fasting. In the case of gross pathology, all the rats in each group were sacrificed humanely by the intraperitoneal administration of pentobarbital 150 mg/kg. Gross pathological changes were recorded for each animal using a hand lens for magnification. i.e., the external surface of the body, body cavities, and their contents with special emphasis on the liver and kidney were examined. Finally, visceral organs were weighted after debridement of the overlying fat and facial coverings (OECD, 2008).

#### Medial lethal dose (LD_50_) determination

In this safety study, the LD_50_ of the fixed oil was determined using probit model analysis under the principle of the guideline and followed procedures applied by essentially identical researchers (Adane et al., 2021). Then, after having the medial lethal dose of the test substance the extended/standing dose for the sub-acute toxicity study was determined by taking 10% of the medial lethal dose as a middle dose (No, 2002).

### Subacute toxicity study

A sub-acute toxicity study was carried out based on the recommendations of OECD 425 guidelines (OECD, 2022). The experimental animals were randomly divided into four groups of 10 rats per group, each group containing five male and five female rats. The fixed oil was administered by oral gavage in doses of 141 mg/kg (G I), 282 mg/kg (G II), and 564 mg/kg (G III) for 28 consecutive days, whilst 2% tween 80 in distilled water was given to rats in the control group (G IV). The doses specified above were based on the acute toxicity study obtained during the oil’s LD50 determination, which was 2818.32 mg/kg. Signs of toxicity and mortality were monitored and recorded regularly, with changes in body weight and daily measurements of food intake. At the end of the study period, animals were fasted overnight, anesthetized using intraperitoneal 150 mg/kg phenobarbital, and blood samples were collected using cardiac puncture. Then, the collected blood was divided into heparinized test tubes for the determination of hematological parameters, and non-heparinized tubes to analyze clinical chemistry. Finally, the visceral organs of both male and female rats were weighed using a standardized calibrated digital analytical balance (METTLER TOLEDO AE 160, Greifensee, Switzerland) after dissection.

### Hematological and clinical chemistry analyses

Ethylenediaminetetraacetic acid (EDTA) was used to process blood samples in test tubes. Hematological parameters were determined on a hematology analyzer (SYSMEX XT-1800i, SYSMEX CORPORATION, Japan). White blood cell count (WBC) with differential counts (Neutrophil, lymphocytes, monocytes, eosinophils, and basophil), red blood cell count (RBC), hemoglobin concentration (HGB), hematocrit (HCT), mean corpuscular volume (MCV), mean corpuscular hemoglobin concentration (MCHC), and platelet count (PLC) were determined. For biochemical analysis, blood samples were allowed to stand for 3 h in plain test tubes for full clotting and centrifuged for 15 min at 5000 rpm using a benchtop centrifuge (Humax-k, Human-GmbH, Germany). The plasma was drained and transferred to other clean vials, and the serum was kept at −20°C until clinical biochemistry measurements were done. The concentrations of alanine aminotransferase (ALT), aspartate aminotransferase (AST), high-density lipoprotein (HDL), low-density lipoprotein (LDL), cholesterol, protein, albumin, urea, and creatinine were automatically determined using DXC 700 AU chemistry analyzer (Beckman Coulter, CA, United States).

### Organ weight measurements, tissue processing, and photomicrography

Body weight was measured, and all experimental animals were sacrificed on day 29. The dissected visceral organs were kept for a few min in 10% formalin to clean any extraneous tissues and weighed with precision balance. The tissue samples from the liver and the kidneys were placed in a test tube with 10% buffered formalin for 24 h and rinsed overnight with tap water. The fixed tissues were then dehydrated and washed with ethanol and xylene, respectively. In addition, it was infiltrated with molten paraffin wax and embedded in paraffin blocks. The blocks were sectioned at a thickness of 4 μm using a Leica rotary microtome (Leica RM2125 RTS, IL, United States). Ribbons of the tissue sections were gently collected using tissue forceps and placed on the surface of a water bath at 30°C–40°C before they were placed over the tissue slide. The slides were then mounted in slide racks and placed overnight in an oven at a temperature of 20°C–40°C to make it easy for the specimens to be fixed on the glass slides. The thin sections then underwent different stages of xylene and alcohol treatment and were stained with hematoxylin and eosin. Then, stained tissue of the liver and the kidney were carefully examined for any signs of histopathological changes using a binocular compound light microscope. Photomicrographs of selected slides from both the treated and the control group were taken using an automated digital photo camera, under a magnification of ×40 and ×20, respectively.

### Data analysis

The data were recorded and entered using EPI Data version 4.60 and exported to SPSS version 25 for analysis. Descriptive statistics; mean, and standard deviation, were done on food intake and body weight, weight gain, organ weight, and amount of food intake. One-way analysis of variance (ANOVA), and independent sample t-test were also conducted for the variables to declare the significant difference between the groups. Tukey’s *post hoc* test was used to confirm the difference among the groups. Finally, p-values <0.05 were considered significantly different between the groups and among groups.

### Ethical consideration

All the experiments were conducted in accordance with internationally accepted laboratory animal use and care guidelines (OECD, 1994), and were approved by the Institutional Review Board of the College of Health Sciences, Addis Ababa University (approval code: 06/2022). Supporting letters were written to the EPHI and AHRI, where the experiments were conducted.

## Results

### Chemical composition of *Lepidium sativum* fixed oil

The chemical constituents identified in the seed oil were as follows: cis-13-Eicosenoic acid (33.4%) was the most abundant fatty acid present in *L. sativum* fixed oil, followed by hexadecanoic acid (20.2%), methyl 11-docosenoate (15.2%), eicosanoic acid (9.8%), methyl stearate (8.0%), and 15-tetracosenoic acid (3.33%). Additionally, benzoic acid (0.013%) and C2-benzene (0.014%) were also detected in the seed oil (Table 1).

**TABLE 1:** Identification of chemical compounds in *L. sativum* fixed oil by GC–MS.

Formula	RT	Area	Percentage (%)	Name
C_8_H_10_	3.214	16,890	0.014	unidentified C2-benzene
C_7_H_8_N_2_O	5.883	15,510	0.013	Benzoic acid, hydrazide
C_9_H_10_O_2_	7.084	46,114	0.038	Methyl phenylacetate
C_10_H_10_O	9.671	50,123	0.041	3-Buten-2-one, 4-phenyl-
C_14_H_30_O	10.006	19,125	0.016	1-Tetradecanol
C_13_H_26_O_2_	11.698	18,657	0.015	Dodecanoic acid, methyl ester
C_15_H_28_O_2_	13.743	22,859	0.018	Myristoleic acid methyl ester
C_15_H_30_O_2_	14.02	329,653	0.272	Myristic acid, methyl ester
C_16_H_30_O_2_	14.863	37,728	0.030	(Z)-10-pentadecenoic acid methyl ester
C_16_H_32_O_2_	15.164	64,365	0.053	Methyl 13-methyltetradecanoate
C_11_H_18_	16.007	19,848	0.016	(R)-(+)-1-(but-3-enyl)-2-methylenecyclohexane
C_17_H_34_O_2_	16.428	24,826,226	20.30	Hexadecanoic acid, methyl ester
C_18_H_36_O_2_	17.306	15,839	0.013	Heptadecanoic acid, methyl ester
C_18_H_34_O_2_	17.381	112,728	0.092	Methyl 9-heptadecenoate or 9-17:1
C_7_H_10_O	18.23	27,945	0.023	1-Ethynylcyclopentanol
C_19_H_38_O_2_	19.091	9,865,017	8.071	Methyl stearate
C_18_H_32_O_2_	19.166	170,805	0.139	9,12-Octadecadienoic acid (Z,Z)-
C_18_H_30_O_2_	19.276	1,552,903	1.270	9,12,15-Octadecatrienoic acid, (Z,Z,Z)-
C_19_H_32_O_2_	19.645	14,237	0.012	9,12,15-Octadecatrienoic acid, methyl ester, (Z,Z,Z)-
C_20_H_40_O_2_	20.46	94,030	0.077	Nonadecanoic acid, methyl ester
C_21_H_38_O_2_	21.499	2,469,230	2.020	cis-11,14-Eicosadienoic acid, methyl ester
C_21_H_40_O_2_	21.632	40,841,578	33.40	cis-13-Eicosenoic acid, methyl ester
C_21_H_42_O_2_	21.938	11,998,005	9.823	Eicosanoic acid, methyl ester
C_18_H_28_O_2_	22.475	17,421	0.0142	(9Z,11E,13S,15Z)-Octadeca-9,11,15-trien-13-olide
C_29_H_50_O	22.966	711,406	0.582	(24R)-Stigmast-5-en-3-beta. -ol
C_20_H_36_O_2_	23.226	98,910	0.0810	Ethyl (9Z,12Z)-9,12-octadecadienoate
C_22_H_44_O_2_	23.342	61,223	0.0500	Heneicosanoic acid, methyl ester
C_15_H_24_O_2_	23.821	66,578	0.0543	(2R,3R,4aR,5S,8aS)-2-Hydroxy-4a,5-dimethyl-3-(prop-1-en-2-yl) octahydronaphthalen-1(2H)-one
C_23_H_44_O_2_	24.462	18,585,166	15.20	Methyl 11-docosenoate
C_23_H_46_O_2_	24.728	3,464,665	2.835	Docosanoic acid, methyl ester
C_25_H_48_O_2_	26.512	4,076,945	3.335	15-Tetracosenoic acid, methyl ester, (Z)-
C_25_H_50_O_2_	26.732	2,503,653	2.048	Tetracosanoic acid, methyl ester

### Acute toxicity

Oral administration of *L. sativum* seed fixed oil to the experimental animals resulted in treatment-related mortality and morbidity. Piloerection, tremor, excessive salivation, and lethargy were observed immediately after treatment with doses of 1,000 mg/kg, 2000 mg/kg, and 5000 mg/kg of the oil but these effects disappeared within 3 h. Moreover, 40% of the animals died at the highest dose of 5000 mg/kg, whilst the low and middle doses caused mortality of 20% of the animals. Using probit analysis, the LD_50_ of the oil was estimated to be 2818.32 mg/kg. The mean difference in the sum of the body weight of controls compared to 2,000 mg/kg and 5000 mg/kg was 13.2 mg and 17.4 mg, respectively. The variation was statistically significant (p = 0.018). Regarding the organ weight, there was a significant variation in the weight of the liver among 2,000 mg/kg, and 5,000 mg/kg compared to the controls (p = 0.01). Compared to controls, the weight of the kidney and spleen showed steady increments in a dose-dependent manner but were not statistically significant (Tables 2, 3).

**TABLE 2:** Effects of different doses of *Lepidium sativum* seed oil on body weight of rats.

	Dose (mg/kg)					
	Control	300	500	1,000	2000	5000
Day 1	184 ± 4.3	181 ± 4.8	183 ± 3.5	184 ± 5.0	181 ± 5.4	183 ± 4.5
Day 7	187 ± 3.6	186 ± 4.3	184 ± 3.1	182 ± 3.3	178 ± 6.4	177 ± 11
Day 14	192 ± 3.1	190 ± 3.9	189 ± 8.1	181 ± 6.4	177 ± 9.8	174 ± 13.2^a^

Data are expressed as mean ± SD, n = 3 for each group (except Group IV, 4, Group V, and VI, each = 3 animals);

^a^ significant at p < 0.05 compared to the control.

**TABLE 3:** Effects of different doses of *Lepidium sativum* seed oil on organ weight of rats.

Organs weighed(g)	Dose (mg/kg)					
	Control	300	500	1,000	2000	5000
Liver	6.6 ± 0.23	6.8 ± 0.21	6.70 ± 0.60	8.0 ± 0.80	8.60 ± 1.30^a^	10.3 ± 0.6^b^
Kidney	1.71 ± 0.05	1.72 ± 0.05	1.72 ± 0.06	1.74 ± 0.07	1.76 ± 0.090	1.82 ± 0.04
Spleen	1.16 ± 0.01	1.16 ± 0.02	1.16 ± 0.04	1.11 ± 0.06	1.17 ± 0.01	1.18 ± 0.11
Heart	1.13 ± 0.04	1.01 ± 0.12	1.15 ± 0.19	1.08 ± 0.18	1.05 ± 0.22	1.16 ± 0.15
Lung	2.36 ± 0.13	2.36 ± 0.16	2.32 ± 0.08	2.52 ± 0.17	2.42 ± 0.23	2.40 ± 0.15
Stomach	2.37 ± 0.30	2.60 ± 0.90	2.37 ± 0.58	2.48 ± 0.13	2.50 ± 0.17	2.34 ± 0.29
Adrenal gland	0.21 ± 0.1	0.18 ± 0.09	0.19 ± 0.08	0.23 ± 0.01	0.17 ± 0.03	0.22 ± 0.09
Thyroid gland	0.76 ± 0.08	0.73 ± 0.11	0.69 ± 0.05	0.77 ± 0.05	0.68 ± 0.03	0.71 ± 0.16

Data are expressed as mean ± SD, n = 3 for each group (except Group IV, 4, Group V, and VI, each = 3 animals);

^a^ significant at P < 0.05 compared to the control, 300 mg/kg, 500 mg/kg, and 1,000 mg/kg;

^b^ significant at p < 0.05 compared to the control and 300 mg/kg, 500 mg/kg, 1,000 mg/kg, and 2000 mg/kg.

### Subacute toxicity studies

#### Effects of the oil on daily food intake and body weight gain

In general, the daily food intake of animals in the control group was higher than those in the treatment group. Comparison of male and female groups showed significant variation in food intake; males consumed more compared to females (p < 0.001). The mean daily food intake of male rats treated with 546 mg/kg showed a statistically significant decrement compared to the control (p = 0.003). Likewise, the mean daily food intake of female rats treated with 546 mg/kg and 282 mg/kg was significantly low compared to controls (p = 0.01 and 0.029, respectively) (Table 4).

**TABLE 4:** Cumulative food intake in grams of rats treated with the fixed oil of *Lepidium sativum* seed.

	Dose (mg/kg)			
	Control	141	282	564
Male	189 ± 1.3	185 ± 3.8	185 ± 3.7	181 ± 1.5^a^
Female	171 ± 1.9	165 ± 3.8	164 ± 3.0	161 ± 4.1^b^

Data are expressed as mean ± SD;

^a^ significant at p < 0.05 compared to controls.

^b^ significant at p < 0.05 compared to controls, and 141 mg/kg.

**FIGURE 1:**
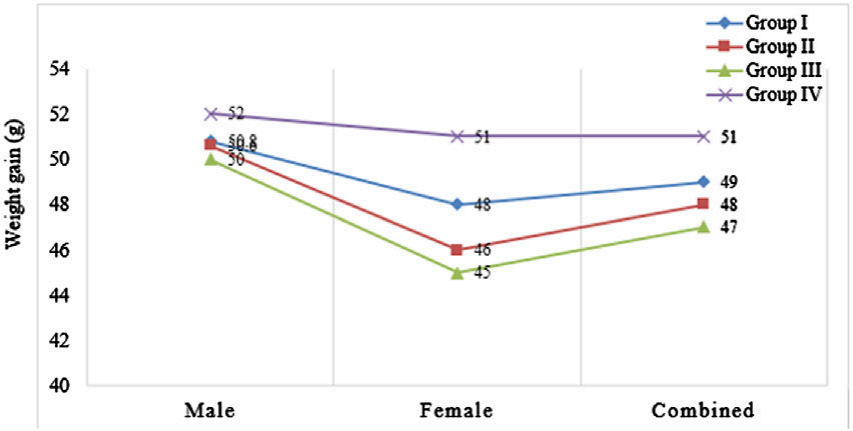
Weight gain of rats treated with the seed fixed oil of *Lepidium sativum* is represented as follows: Group I (141 mg/kg) is indicated by diamonds on the Y-axis, Group II (282 mg/kg) is represented by squares on the Y-axis, Group III (564 mg/kg) is shown with triangles on the Y-axis, and Group IV, which served as a control group, was treated with 2% Tween 80 in distilled water and is marked with an “X” on the Y-axis).

As shown in Figure 1, there was a significant variation in body weight gain in the control group compared to the 282 mg/kg and 564 mg/kg treated groups (p = 0.005). Body weight gain of rats treated with 282 mg/kg fixed oil was significantly lower compared to the control group (p = 0.023). In addition, the highest dose (564 mg/kg) treated groups had significantly lower mean body weight gain compared to the control (p = 0.006) (Figure 1).

#### Effects of the oil on organ weight

In both male and female rats, the weights of the liver and kidneys showed a steady increment in the higher-dose treatment groups compared to the control. In male rats, the weight of the right kidney (p = 0.001) and the left kidney (p < 0.01) were significantly higher in the 564 mg/kg treated groups compared to the control and 141 mg/kg groups. Similarly, in female rats, the right (p = 0.031) and left (p = 0.04) kidneys were significantly higher in weight than the control rats. In both male and female rats, the liver was larger in the treatment groups in a dose-dependent manner compared to the control. Both male and female rats treated with 546 mg/kg fixed oil had significantly larger livers than the control, 141 mg/kg, and 282 mg/kg treated groups (p < 0.001). On the other hand, the oil does not seem to affect the weight of the other organs studied (Table 5).

**TABLE 5:** Effect of 28 days oral administration of *Lepidium sativum* seed fixed oil on organ weight of Wistar albino rats.

Organ weighed (g)	Dose mg/kg							
	Male				Female			
	Control	141	282	564	Control	141	282	564
Liver	9.10 ± 0.19	10.0 ± 0.60	10.9 ± 0.70	14.5 ± 0.70^c^	8.00 ± 0.0.78	9.90 ± 0.4	10.3 ± 0.40	17.0 ± 2.30^c^
Rt kidney	0.96 ± 0.01	0.96 ± 0.2	1.0 ± 0.05	1.10 ± 0.07^b^	0.78 ± 0.05	0.79 ± 0.01	0.80 ± 0.03	0.83 ± 0.20^a^
Lt kidney	0.93 ± 0.01	0.94 ± 0.01	0.99 ± 0.03	1.00 ± 0.05^b^	0.77 ± 0.02	0.78 ± 0.01	0.79 ± 0.02	0.81 ± 0.01^a^
Spleen	1.24 ± 0.05	1.21 ± 0.07	1.24 ± 0.06	1.27 ± 0.05	1.12 ± 0.07	1.16 ± 0.02	1.11 ± 0.06	1.18 ± 0.01
Stomach	2.80 ± 0.83	4.0 ± 0.43	3.37 ± 0.30	2.64 ± 0.70	2.46 ± 0.30	2.34 ± 0.50	2.42 ± 0.34	2.38 ± 0.37
Heart	1.29 ± 0.01	1.27 ± 0.18	1.27 ± 0.08	1.30 ± 0.08	1.20 ± 0.05	1.19 ± 0.07	1.20 ± 0.05	1.13 ± 0.05
Rt lung	1.22 ± 0.05	1.18 ± 0.04	1.18 ± 0.10	1.22 ± 0.04	0.84 ± 0.06	0.91 ± 0.04	0.87 ± 0.07	0.89 ± 0.04
Lt lung	1.18 ± 0.10	1.14 ± 0.10	1.18 ± 0.10	1.22 ± 0.08	0.87 ± 0.07	0.88 ± 0.05	0.86 ± 0.01	0.87 ± 0.07
Adrenal gland	0.31 ± 0.07	0.35 ± 0.04	0.35 ± 0.10	0.29 ± 0.08	0.22 ± 0.04	0.21 ± 0.08	0.25 ± 0.03	0.25 ± 0.04
Thyroid gland	1.15 ± 0.12	1.26 ± 0.02	1.28 ± 0.04	1.20 ± 0.08	0.77 ± 0.10	0.73 ± 0.11	0.77 ± 0.10	0.71 ± 0.16

Data are expressed as mean ± SD; *Significant p-value when males and females are grouped.

^a^ significant at p < 0.05 compared to controls.

^b^ Significant difference of the highest dose compared to controls, and141 mg/kg.

^c^ Significant difference of the highest dose group (546 mg/kg), compared to282 mg/kg,141 mg/kg, and control groups.

#### Effect of the oil on hematological parameters of rats

The number of white blood cells (WBCs) was decreased in all treatment groups compared to control in a dose-dependent manner (p < 0.001). Male rats treated with 546 mg/kg had the lowest number of WBCs compared to the control and 141 mg/kg treated groups (p < 0.001). Similarly, female rats of the 564 mg/kg treated group had fewer WBC counts than the control rats (p < 0.04). Animals treated with all doses of the fixed oil showed a significant decrement in neutrophils and monocyte counts. Other hematological parameters like HGB, MCHC, and MCV showed variations among the controls and treatment groups but they were not significant (Table 6).

**TABLE 6:** Effect of *Lepidium sativum* seed fixed oil on mean hematological parameters of rats after28−day repeated oral doses.

Parameter	Dose mg/kg							
	Male				Female			
	Control	141	282	564	Control	141	282	564
WBC × 10^3^/μL*	7.3 ± 0.32	6.8 ± 0.28	6.9 ± 0.56	6.3 ± 0.32^a^	6.8 ± 0.75	6.0 ± 0.20	6.2 ± 0.27	5.9 ± 0.48^a^
RBC ×106/μL	7.4 ± 0.69	7.2 ± 0.22	7.1 ± 0.80	7.18 ± 0.81	6.8 ± 0.40	6.7 ± 0.19	6.5 ± 0.33	6.46 ± 0.26
HGB (g/dL)	15.6 ± 0.77	15.9 ± 0.60	16.1 ± 0.32	16.2 ± 0.56	14.5 ± 0.80	15.1 ± 0.5	15.3 ± 0.6	15.2 ± 0.29
HCT (%)	36.9 ± 1.3	37.7 ± 0.70	37.7 ± 0.65	37.6 ± 0.91	35.2 ± 1.3	36.0 ± 0.9	36.4 ± 1.1	36.1 ± 0.58
MCV (FL)	59 ± 0.78	61 ± 1.60	60.6 ± 1.15	60.34 ± 1.8	58.7 ± 2.6	58.4 ± 1.2	56.5 ± 1.5	56.2 ± 2.5
MCH (pg)	22.4 ± 0.8	23 ± 1.20	23.1 ± 1.3	24.1 ± 0.50	21 ± 0.64	20.6 ± 0.7	21.9 ± 0.66	22.1 ± 1.2
MCHC (g/dL)	38.1 ± 0.1	38.3 ± 0.7	38.6 ± 0.9	39.1 ± 0.68	36 ± 0.84	36.1 ± 0.8	36.1 ± 0.55	37.1 ± 0.44
PLT ×103/μL	511 ± 23.0	486 ± 28.00	481 ± 35.00	475 ± 45.0	505 ± 41.00	491 ± 33.80	487 ± 26.10	485 ± 31.00
RDW (%)	16.7 ± 0.5	16.6 ± 0.55	16.9 ± 0.23	16.8 ± 0.35	16 ± 0.22	15.7 ± 0.35	15.8 ± 0.24	15.6 ± 0.24
RDW-SD (%)	28.7 ± 0.71	28.3 ± 0.43	29.1 ± 1.0	29.6 ± 0.27	25.6 ± 0.7	26.5 ± 0.42	26.8 ± 1.2	26.6 ± 0.68
NEUT (%)	8.1 ± 0.43	7.9 ± 0.30	7.8 ± 0.27	7.3 ± 0.23^a^	6.0 ± 0.04	5.8 ± 0.11	5.7 ± 0.14	5.4 ± 0.3^b^
LYMPH (%)	82 ± 0.60	81.5 ± 1.10	82.4 ± 0.60	82.6 ± 1.0	79.5 ± 1.60	77 ± 1.30	77.2 ± 1.50	78.6 ± 2.10
MONO (%)*	2.86 ± 0.08	2.74 ± 0.12	2.73 ± 0.03	2.64 ± 0.04^a^	2.6 ± 0.02	2.54 ± 0.1	2.47 ± 0.1^c^	2.52 ± 0.12
EO (%)	1.0 ± 0.17	1.10 ± 0.11	1.2 ± 0.15	1.16 ± 0.15	1.1 ± 0.16	1.0 ± 0.17	1.0 ± 0.16	1.0 ± 0.13
BASO (%)	0.26 ± 0.04	0.39 ± 0.15	0.29 ± 0.08	0.32 ± 0.09	0.35 ± 0.07	0.3 ± 0.07	0.39 ± 0.04	0.35 ± 0.07

Data are expressed as mean ± SD.

*Significant p-value when males and females are grouped.

^a^ Significant variation between 564 mg/kg treated group compared to controls.

^b^ Significant variation between 564 mg/kg treated compared to controls, and 141 mg/kg.

^c^ Significant variation between 282 mg/kg treated compared to control groups; Where p-value significant at <0.05; PLT, platelets; RBC, red blood cells; WBC, white blood cells; MCV, mean corpuscular volume; MCH, mean corpuscular hemoglobin; MCHC, mean corpuscular hemoglobin concentration; RDW, red cell distribution width; HCT, hematocrit; HGB, hemoglobin.

#### Effects of the oil on the biochemical parameters of rats

Clinical chemistry results showed significant variation in values between treated and control groups. A significantly higher level of AST was observed in the highest-dose treated group compared to the control (p = 0.001). Similarly, ALT and ALP were elevated in those treated with 564 mg/kg compared to the control group (p = 0.001, and p = 0.004) respectively (Table 7).

**TABLE 7:** Biochemical profile of rats treated with the fixed oil of *Lepidium sativum* seed.

Parameters	Dose mg/kg							
	Male				Female			
	Control	141	282	564	Control	141	282	564
ALT (U/L)*	83.9 ± 3.0	94.0 ± 15.8	97.0 ± 4.6	100.0 ± 4.76^a^	82.6 ± 6.1	85.8 ± 4.3	85.6 ± 3.3	94.4 ± 7.6^a^
AST (U/L)*	238 ± 11.7	244 ± 5.8	253 ± 7.2	284 ± 46.3^a^	211 ± 18.0	225 ± 13.0	232 ± 12.0	252 ± 34.0^a^
ALP (U/L)*	89.6 ± 15.6	87.4 ± 5.2	94.4 ± 9.6	116.4 ± 27.0	72.6 ± 4.0	78.0 ± 10.8	81.6 ± 4.8	95.6 ± 21.0^a^
Albumin (g/dL)	3.32 ± 0.20	3.20 ± 0.11	3.40 ± 0.05	3.34 ± 0.13	3.26 ± 0.05	3.18 ± 0.08	3.30 ± 0.11	3.22 ± 0.11
Protein (g/dL)	6.9 ± 0.13	7.1 ± 0.54	7.1 ± 0.35	7.0 ± 0.47	5.7 ± 0.50	5.6 ± 0.77	5.96 ± 0.27	5.91 ± 0.28
Cholesterol (mg/dL)	72.4 ± 7.6	67.5 ± 6.6	66.4 ± 6.3	63.4 ± 4.3	74.2 ± 4.0	72.8 ± 4.8	68 ± 6.4	67.6 ± 5.4
HDL (mg/dL)	56.4 ± 9.8	63.4 ± 6.1	62.4 ± 10.5	70.0 ± 8.8	43.4 ± 8.6	48.6 ± 6.0	48.6 ± 6.8	56.2 ± 5.3
LDL (mg/dL)	27.8 ± 5.4	25.0 ± 4.3	25.4 ± 4.3	22.4 ± 6.0	24.0 ± 5.0	21.0 ± 4.60	22.8 ± 6.0	19.6 ± 5.0
Urea (mg/dL)	37.4 ± 3.8	41.8 ± 2.9	41.0 ± 4.3	43.0 ± 1.6	34.6 ± 3.9	37.0 ± 2.90	39.4 ± 3.60	40.0 ± 3.00
Creatinine (mg/dL)	0.50 ± 0.17	0.52 ± 0.26	0.50 ± 0.14	0.71 ± 0.11	0.32 ± 0.20	0.36 ± 0.19	0.43 ± 0.10	0.52 ± 0.11

* Significant p-value when males and females are grouped.

^a^ significant variation between1000 mg/kg treated and control groups when males and females are treated separately; Where, p-value significant at <0.05; ALP: alkaline phosphatase, ALT: alanine aminotransferase, AST: aspartate aminotransferase, HDL: High-density lipoprotein, LDL: Low-density lipoprotein, mg: milligram, dL: deciliter.

#### Effects of the oil on gross morphology and histopathology of organs

**FIGURE 2:**
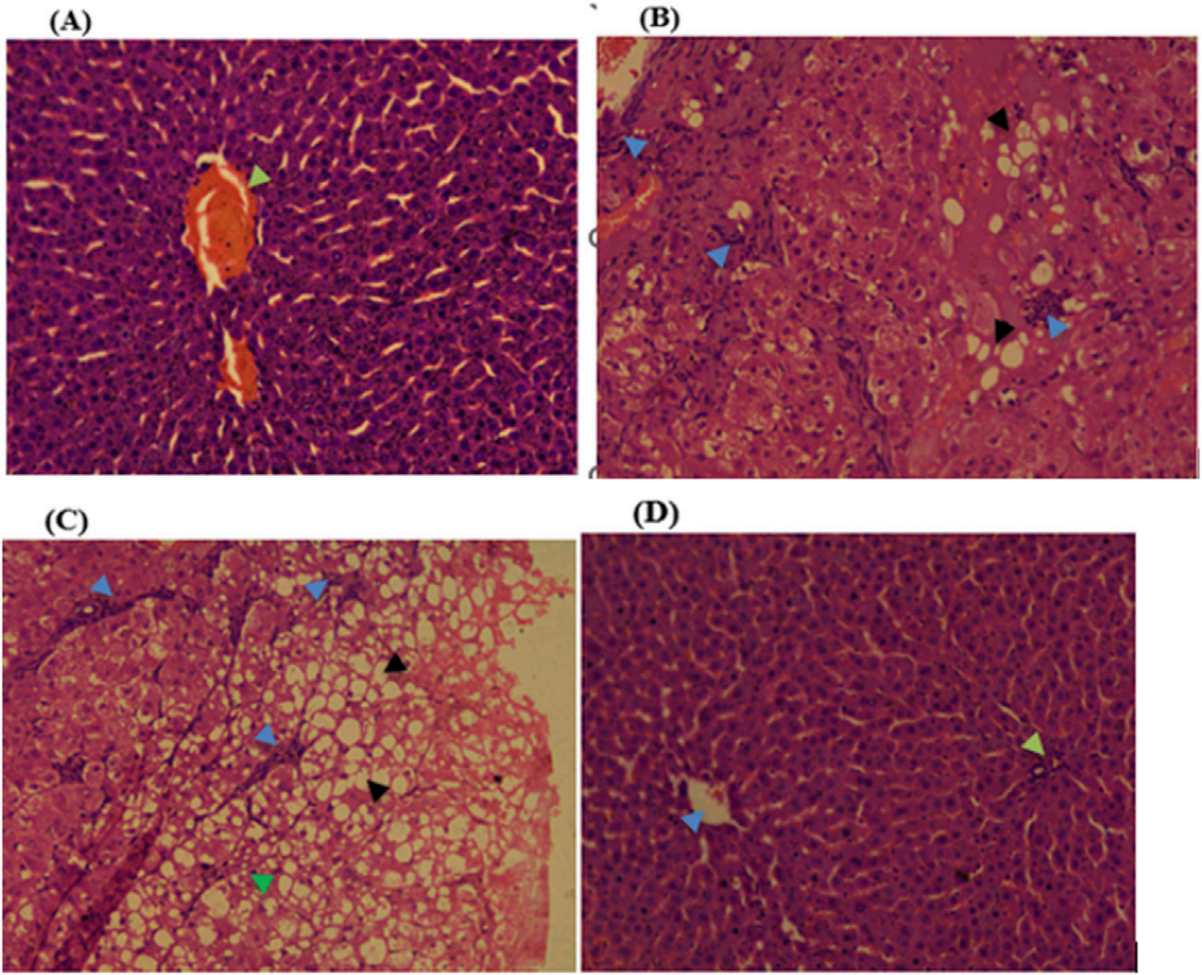
Photomicrograph of liver in the female rats revealed distinct histopathological changes at various dose levels of the fixed oil of *Lepidium sativum*, compared to control rats. **(A)** At a dose of 141 mg/kg, the liver showed a central vein (indicated by green arrowheads). **(B)** At 282 mg/kg, the observations included focal macrovesicular vacuolations (noted as hollow whitish cells) and focal leukocyte infiltrations (indicated by a blue arrowhead). **(C)** At 564 mg/kg, the liver displayed diffuse macrovesicular vacuolations (depicted as whitish hollow/empty cells), microvesicular vacuolations (shown by a green arrowhead), and focal leukocyte infiltration (indicated by a blue arrowhead). **(D)** The control group, treated with Tween 80%, showed a central vein (indicated by blue arrowheads) and a portal triad (marked with green arrowheads).

**FIGURE 3:**
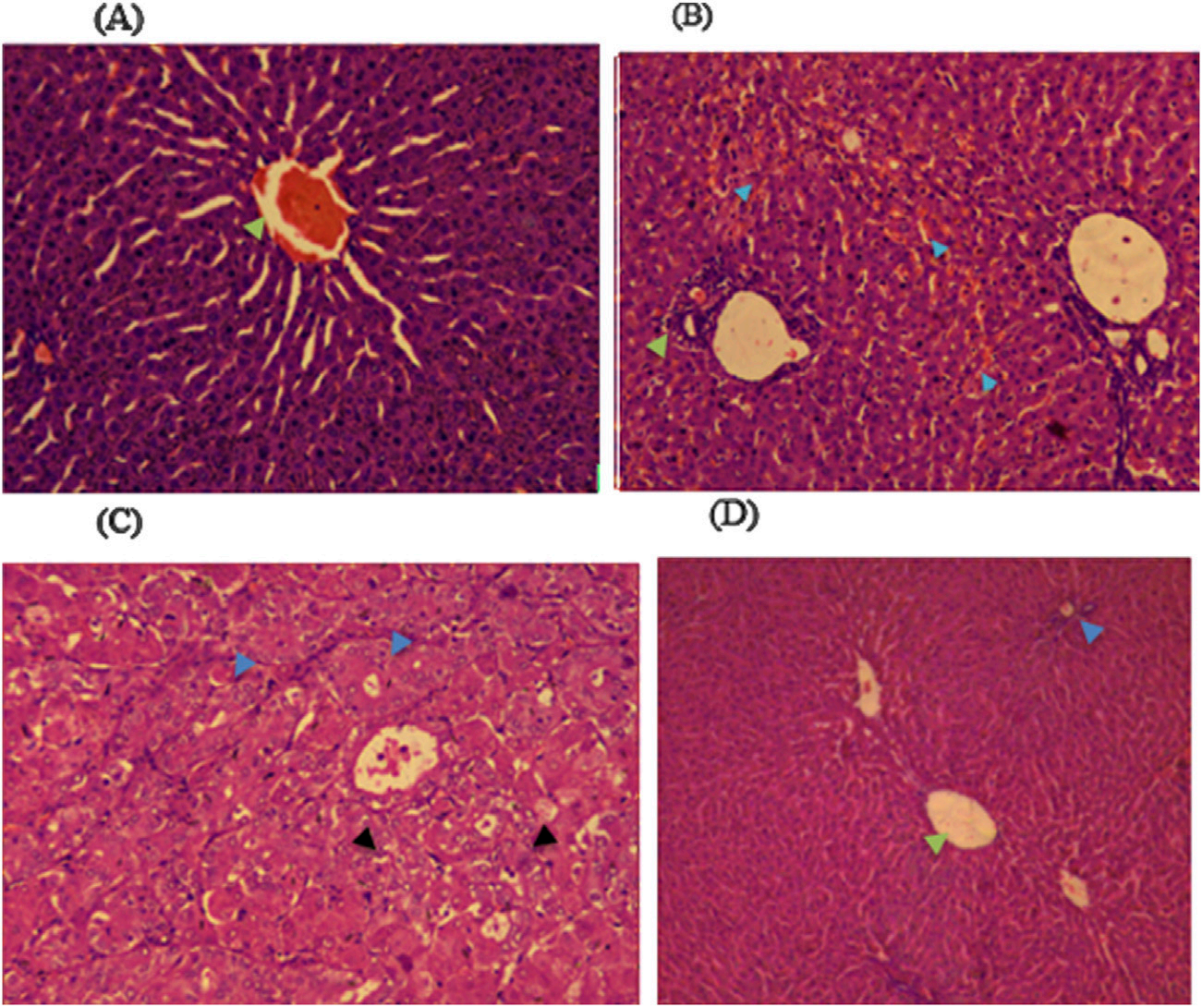
Photomicrograph of liver in the male rats revealed distinct histopathological changes among the different dose levels of *Lepidium sativum* fixed oil in treated rats compared to the control group. **(A)** In the group treated with 141 mg/kg, the central vein exhibited prominent sinusoidal striations (green arrowhead). **(B)** The 282 mg/kg treated group displayed a proliferation of bile ducts (green arrowhead) and sinusoidal congestion (blue arrowhead). **(C)** In the 564 mg/kg treated group, there is a marked swelling of hepatocytes (blue arrowhead) along with microvesicular vacuolations (black arrowhead). **(D)** The control group demonstrated a clear central vein (green arrowhead) and an intact portal triad (blue arrowhead).

**FIGURE 4:**
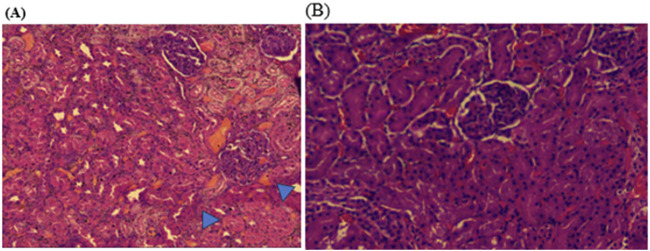
Photomicrograph of kidney in the female rats revealed histopathological differences between the highest dose treated group (564 mg/kg) and the control rats. **(A)** The 564 mg/kg treated group exhibited minimal parenchymal swelling (blue arrowheads), while **(B)** the control group showed normal kidney architecture.

**FIGURE 5:**
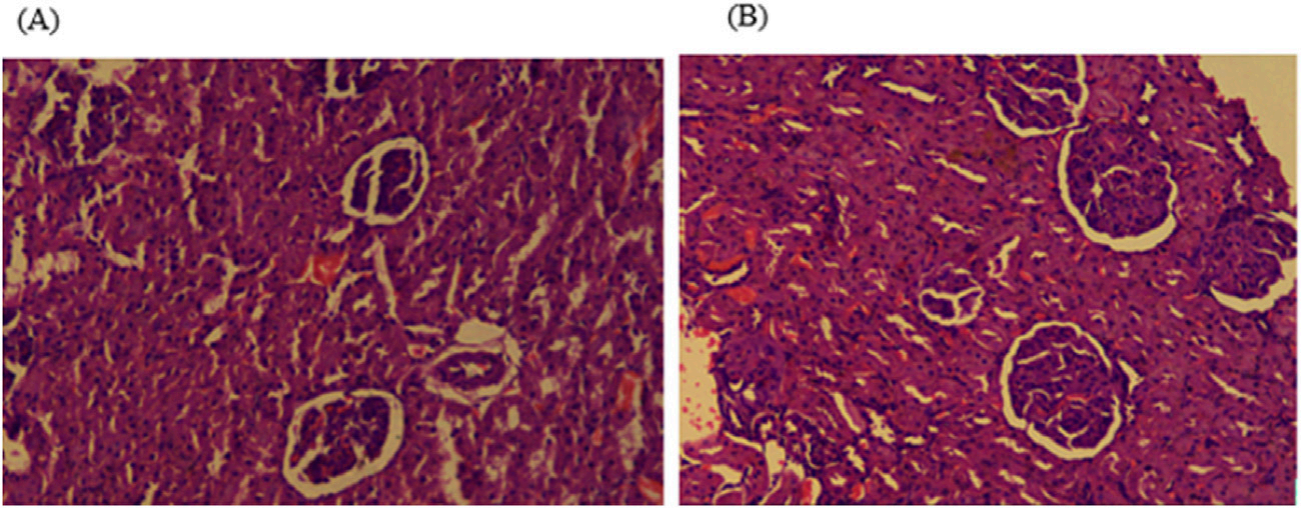
Photomicrograph of kidney in the male rats showed no obvious histological differences among the highest dose treated (564 mg/kg) and control rats; **(A)** 564 mg/kg treated group, and **(B)** control group kidney.

Histopathological examinations of organs in treatment groups (282 mg/kg, and 564 mg/kg) revealed extensive micro and microvesicular vacuolation of hepatocytes, focal leukocytic infiltration, sinusoidal congestion, and occasional fatty change in the liver compared to the control group. Central venous thrombosis and parenchymal tissue architectural disarray were observed in a 564 mg/kg treated group of both male and female rats. Liver parenchyma in the form of sinusoidal dilation and congestion was observed in female rats treated with 564 mg/kg for consecutive 28 days. In addition, female rats treated with high doses of the oil showed focal peri-portal fibrosis and peri-portal necrosis. They also showed bile duct proliferation, when compared to the control group (Figures 2, 3) and (Table 8). However, histopathological variation between the treatment and the control groups was not observed except minimal parenchymal swelling was observed in female rats treated with 564 mg/kg of the oil (Figures 4, 5).

**TABLE 8:** Frequency of liver pathologic findings in rats treated with the fixed oil of *Lepidium sativum* seed.

Histopathologic parameters	Dose mg/kg				
		Control	141	282	564
Micro vesicular vacuolation	Male	—	1	1	4
	Female	—	—	2	4
Macro vesiculation vacuolation	Male	—	1	1	4
	Female	—	—	1	5
Leucocyte infiltration	Male	1	—	1	2
	Female	—	—	2	2
Periportal necrosis	Male	—	—	—	1
	Female	—	—	1	2
Bile duct proliferation	Male	—	—	—	1
	Female	—	—	1	2
Central venous thrombosis	Male	—	—	1	2
	Female	1	—	—	1
Sinusoidal congestion	Male	—	—	—	2
	Female	—	—	—	2

## Discussion

Experimentation with animals makes it possible to learn about the toxic potential of drugs and other chemicals (Zbinden, 1991). Findings in animal toxicology studies generally apply to humans, although responses of laboratory animals and humans to chemicals may differ qualitatively and quantitatively (Council, 2004). Hence, it has become a primary necessity to evaluate the toxic nature of any compound before accepting it as a pharmaceutical or nutraceutical excipient (Malik et al., 2022). The current study presented the first comprehensive evaluation of *L. sativum* fixed oil seed safety in Ethiopia. It was revealed that the LD_50_ of *L. sativum* fixed oil was 2818.32 mg/kg, putting the plant in GHS Category 5 and Class III in the WHO classification system, which makes it a slightly hazardous product (Ta et al., 2011).

The 28-day repeated-dose toxicity study revealed that *L. sativum* seed fixed oil causes significant alterations in hematological and clinical chemistry parameters. The oil caused derangements in gross morphometry and histopathology of the liver in the higher-dose treated groups. Daily food intake steadily dropped in the 564 mg/kg dose groups compared to the control. The result was comparable to the study conducted by Adam et al. (Westphal, 2017), where higher-dose groups of rats developed significant weight reduction contrary to their counterparts. The possible reason might be the direct gastrointestinal irritation effect of the fixed oil, which interferes with regular food consumption (Liu et al., 2008), as some people who use *L. sativum* seed oil as a cooking medium experience symptoms of indigestion (Singh and Paswan, 2017). Therefore, a comprehensive study of dietary optimization considering *ad libitum* groups is necessary to verify the exact cause of decreased food intake. The fixed oil showed a significant decline in body weight of the treatment groups of both sexes compared to the controls. The result is supported by the study conducted by Bafeel and Ali (2009). The decrease in body weight observed in the current study might be the presence of linoleic acid, which is the main ingredient of the fixed oils of *L. sativum* (Solomon et al., 2016). It has been reported that supplementation of linoleic acid improves the ratio of lean body mass to fat, resulting in decreased visceral body fat and increased body muscle mass (DeLany et al., 1999). In addition, *L. sativum* has anti-nutritional compounds such as phytin, phosphorus, and oxalates, which interfere with the absorption of nutrients in the body and metabolic processes (Singh and Paswan, 2017).

There was a steady increment in the weight of the liver among treatment groups in a dose-dependent manner. The result was comparable with the study conducted by Bafeel and Ali (2009), but not similar to the study conducted by Datta et al. (2011). It might be due to an adaptive response as the liver is the main site of xenobiotic metabolism, which results in cellular hyperplasia and hypertrophy due to the increased functional load. However, it was not possible to identify whether the enlargement is due to additive growth, or regenerative growth. Strangely, liver weight was significantly higher in female rats than in male rats of higher dose groups. This can be explained by males being more toxic resilient than females (Vahter et al., 2007), as mRNA expression differences between males and females, and differences in protein levels and regulation of activity via post-translational modifications (Nicolson et al., 2010). Additionally, males exhibited significantly higher levels of microsomal P450 content and greater NADPH-CYP oxidoreductase activity, with many P-450 enzyme activities also being higher in males (Guo et al., 1993). The predominance of antioxidant enzymes in male rats compared to females may contribute to their greater resilience to toxins. CYP1A2 is a major enzyme in the liver responsible for the metabolism of various clinically important drugs, as well as several endogenous compounds like arachidonic acids and steroids. Research has shown that the activity of microsomal CYP1A2, measured through 7-ethoxyresorufin O-dealkylase activity, tends to be higher in males than in females. Furthermore, males may also exhibit greater activity compared to females for other enzymes such as CYP2E1, the drug efflux transporter P-glycoprotein (P-gp/MDR1), and certain isoforms of glucuronosyltransferases and sulfotransferases (Meibohm et al., 2002; Zhou et al., 2009; Parkinson et al., 2004)

Kidney weight was significantly enlarged in higher dose treatment groups compared to the control. The finding was comparable with the study conducted by Westphal (2017), where *L. sativum* seed treatment resulted in increased weight of the kidney compared to control. The increase in the weight of the kidney could be due to swelling resulting from the toxicant injury with concomitant derangement of membrane permeability, enzyme activity, and transport characteristics of the cell (Schnellmann, 2001).

The extent of the toxic effect of drugs and/or plant extracts can be determined by the evaluation of hematological parameters (OA et al., 2002). In this regard, the number of WBCs was significantly decreased in higher dose groups compared to the control. The result was comparable with the study conducted by Adam (1999), but not the studies conducted by Bafeel and Ali (2009) and Datta et al. (2011). The decrease in total WBC count in higher dose treatment groups could be due to malfunctioning of the hematopoietic system caused due to exposure to the bioactive compound found in the oil like thiocyanate, and benzoic acid which could disrupt the cell cycle resulting in DNA fragmentation of cells and cellular apoptosis (Mahassni and Al-Reemi, 2013; Shu et al., 2016). Additionally, dietary inclusion of benzoic acid supplementation reduced the white blood cell count and globulin levels (Shu et al., 2016). Major types of blood cells such as leukocytes are susceptible to benzene toxicity. The most characteristic systemic effect resulting from intermediate and chronic benzene exposure is the arrested development of blood cells. The effect is characterized by a reduction of all cellular elements in the peripheral blood and bone marrow, leading to fibrosis, an irreversible replacement of bone marrow (Shahnawaz et al., 2013). The total leukopenia and differential count reduction could also be associated with concomitant bone marrow depletion, which is attributable to nutritional deficiency and or stress in higher-dose treatment groups (Moriyama et al., 2008). Moreover, the reduced number of neutrophils and other components of the differential counts could be attributable to xenobiotic-induced agranulocytosis which may involve a sudden depletion of circulating neutrophils concomitant with exposure that persists as long as the agent or its metabolites are in circulation (Hall, 2020). This effect might be linked to the prolonged administration of the substance. However, the short-term effects of the fixed oil on blood chemistry remain unknown. Therefore, it would be beneficial to compare both the short-term and long-term effects of the seed extract, taking into account the differences in inflammatory responses associated with varying durations of exposure.

Several biochemical indices can be measured to determine the safety of medicinal plants (Dzoyem et al., 2014). In this study, serum levels of ALT and AST were elevated in rats treated with 564 mg/kg of the oil. This is comparable with the studies conducted by Bafeel and Ali (2009), Datta et al. (2011), and Adam (1999). It might be due to the presence of isothiocyanate in the oil (Burow et al., 2007), which are toxic defensive chemical used by herbs against a variety of organisms (Rask et al., 2000). Elevation of the above enzymes could be due to the fact that ALT & AST enzymes are cytosolic marker enzymes reflecting hepatocellular necrosis as they are released into the blood after cell membrane damage (Andallu and Vardacharyulu, 2001). However, the above level cannot be used to predict either the type of lesion, or whether cell damage is reversible (leakage) or irreversible (frank necrosis) (Dzoyem et al., 2014). The extent of the damage along with the liver histopathological results can be predicted. ALP was significantly elevated in higher-dose treatment groups compared to their controls. The result was comparable with the studies reported by Datta et al. (2011) and Bafeel and Ali (2009). The reason might be due to the body’s inability to excrete it through bile as a result of disturbed bile flow. Apart from the above, urea is the most frequently determined clinical index for estimating renal function depending on urea concentration in the serum (Gowda et al., 2010). Even though it was not statistically significant, serum urea was steadily increased in higher-dose treatment groups compared to the control.

The oil showed toxic effects on the liver microarchitecture represented by periportal hepatocyte necrosis, and focal mononuclear cell infiltration in the 564 mg/kg treated rats. This is accompanied by the biochemical alterations and gross morphometric findings of treatment groups discussed in this study and comparable with the study conducted by Bafeel and Ali (2009). This toxic change might be due to the presence of isothiocyanate which is one of the major constituents of *L.sativum* seed (Burow et al., 2007; Burow and Wittstock, 2009). Focal macro and microvesicular vacuolation, bile duct proliferation, and fatty changes were observed in 282 mg/kg, and 564 mg/kg treated groups. The result was comparable with the study conducted by Bafeel and Ali (2009). This might be due to a considerable increase in liver cholesterol and triglyceride content with a concomitant drop in the amount of glycogen due to the effects of isothiocyanate (Okulicz et al., 2005; Okulicz, 2010). In this regard, macro and microvesicular vacuolation of hepatocytes could be due to glycogen depletion and concomitant increase in liver triglyceride/lipid content. Bile duct proliferation could be explained by metabolic changes induced by the presence of isothiocyanate in the oil. The parenchyma showed vascular congestion of the central vein in the 564 mg/kg treatment groups. This could be due to anoxia of the central region of the liver lobules secondary to obstruction of the blood flow through the sinusoids. This obstruction can arise from vacuolation of the parenchymal cells which leads to a diminished vascular bed within the liver (Andrews and Maegraith, 1948). In histopathological analysis of the kidney, no significant parenchymal lesion was observed except minimal parenchymal swelling was shown in the 564 mg/kg treated groups. This result was consistent with the biochemical findings and comparable with the study conducted by Westphal (2017).

## Conclusion and recommendations

Oral administrations of the fixed oil of *L sativum* appeared to be toxic to rats. It produced overt signs of toxicity in both male and female rats during single and repeated dose studies. The oil showed undesirable effects on body growth, organ weights, and hematological and biochemical parameters that were supported by gross and histopathologic examinations of major organs. Therefore, it is highly essential to create awareness among the Ethiopian public who use the seeds for medicinal purposes and/or consume the oil as edible oil about the possible health hazards that they may pose. Besides, it would be interesting to conduct developmental toxicity studies of the oil as it is commonly used by all categories of people including pregnant women.

## Data Availability

The original contributions presented in the study are included in the article/supplementary material, further inquiries can be directed to the corresponding author.

## Abbreviations

ALP, Alkaline phosphatase; ALT, Alanine aminotransferase; ANOVA, One-way analysis of variance; AST, Aspartate Aminotransferase; GHS, Globally Harmonized System of Classification and Labeling of Chemicals; L. sativum, Lepidium sativum; OECD, Organization for Economic Cooperation and Development.
